# A Possible Role of HMGB1 in DNA Demethylation in CD4^+^ T Cells from Patients with Systemic Lupus Erythematosus

**DOI:** 10.1155/2013/206298

**Published:** 2013-09-03

**Authors:** Yaping Li, Chenghui Huang, Ming Zhao, Gongping Liang, Rong Xiao, Susan Yung, Tak Mao Chan, Qianjin Lu

**Affiliations:** ^1^Department of Dermatology and Epigenetic Research Center, Key Laboratory of Diabetes Immunology of Ministry of Education, Second Xiangya Hospital, Central South University, Changsha, Hunan 410011, China; ^2^Department of Oncology, Third Xiangya Hospital, Central South University, Changsha, Hunan 410011, China; ^3^Department of Medicine, The University of Hong Kong, Hong Kong

## Abstract

The aberrant activity of CD4^+^ T cells in patients with systemic lupus erythematosus (SLE) is associated with DNA hypomethylation of the regulatory regions in CD11a and CD70 genes. Our previous studies demonstrated that Gadd45a contributes to the development of SLE by promoting DNA demethylation in CD4^+^ T cells. In this study, we identified proteins that bind to Gadd45a in CD4^+^ T cells during SLE flare by using the method of co-immunoprecipitation and mass spectrometry, High mobility group box protein 1 (HMGB1) is one of identified proteins. Furthermore, gene and protein expression of HMGB1 was significantly increased in SLE CD4^+^ T cells compared to controls, and HMGB1 mRNA was correlated with CD11a and CD70 mRNA. A significant, positive correlation was found between HMGB1 mRNA and SLEDAI for SLE patients. Our data demonstrate that HMGB1 binds to Gadd45a and may be involved in DNA demethylation in CD4^+^ T cells during lupus flare.

## 1. Introduction

Systemic lupus erythematosus (SLE) is a prototype autoimmune disease that affects multiple organ systems. The etiology and pathogenesis of SLE remain to be fully defined. Recent studies have shown that T-cell DNA demethylation plays an important role in the pathogenesis of SLE [1]. We previously demonstrated that inhibition of DNA methylation in T cells resulted in the demethylation of regulatory sequences and increased gene expression of CD11a (ITGAL) and CD70 (TNFSF7) [2–4]. Increased expression of these genes results in T-cell autoreactivity and autoantibody production by the B lymphocyte lineage. The molecular mechanisms that mediate gene demethylation in SLE CD4^+^  T cell are not fully understood, but it is possible that growth arrest and DNA damage-inducible gene (Gadd45a/Gadd45) may contribute since increased expression of Gadd45a gene has been shown to repress DNA methylation, and factors that induce lupus flare such as ultraviolet have been shown to promote DNA demethylation in SLE CD4^+^  T cells [5–7]. 

In this study, we identified protein(s) that bind to Gadd45a, assessed their gene and protein expression and their association with CD11a, CD70 mRNA, and lupus disease activity. HMGB1 was identified as a major protein that bind to Gadd45a, which may contribute to DNA demethylation.

## 2. Materials and Methods

### 2.1. Patients and Control Subjects

Ten SLE patients (28.50 ± 10.23 years of age) were recruited from the outpatient clinics and inpatient Department of Dermatology at the Second Xiangya Hospital, Central South University. All patients fulfilled at least 4 of the SLE classification criteria of the American College of Rheumatology [8]. Disease activity was quantified by the SLE Disease Activity Index (SLEDAI) [9], and the mean SLEDAI score of patients recruited into this study was 7.20 ± 1.93 (Table 1). Age- and sex-matched healthy controls (25.80 ± 6.89 years of age) were recruited from the medical staff at the Second Xiangya Hospital. This study was approved by the human ethics committee of the Second Xiangya Hospital. 

### 2.2. Isolation of CD4^+^  T Cells

Peripheral blood mononuclear cells were isolated from heparin-anticoagulated whole blood using Ficoll-Hypaque density gradient centrifugation (Shanghai Hengxin Chemical Reagent Co., Ltd., Shanghai, China), and CD4^+^  T cells were isolated by positive selection using Miltenyi beads, according to the manufacturer's instructions (Miltenyi, Germany). The purity of the enriched subsets was validated by flow cytometry and was >95%.

### 2.3. Coimmunoprecipitation and LS-MS/MS

CD4^+^  T cells were solubilized with cell lysis buffer (KeyGEN, China). Aliquots of CD4^+^  T-cell lysates were incubated overnight at 4°C with rabbit anti-human Gadd45a antibody (Santa Cruz Biotechnology, USA) or isotype-matched rabbit IgG (Beyotime, China) prior to the absorption with protein A/G PLUS-agarose beads (Millipore, USA) at 4°C for 1 hour. Proteins that bound to Gadd45a were centrifuged and eluted. Immunoprecipitated samples were incubated with 2 × SDS electrophoresis sample buffer at 95°C for 5 min and subjected to SDS-PAGE followed by staining with Coomassie blue. Bands of interest were excised, and sent to BGI (Shenzhen, China) for identification using LS-MS.

### 2.4. Western Blot

Aliquots of CD4^+^  T-cell proteins were denatured, separated by SDS-PAGE, and transferred to PVDF membranes (Amersham Bioscience, GE Healthcare Company, USA). The membranes were incubated overnight at 4°C with rabbit anti-human HMGB1 polyclonal antibody (diluted 1 : 500, Abcam, USA) followed by incubation with horseradish peroxide-conjugated mouse anti-rabbit antibody (diluted 1 : 3000, Santa Cruz, USA). Bands were detected with Pierce chemiluminescence detection system (Thermo Scientic, Rockford, IL, USA). All bands were normalized to *β*-actin.

### 2.5. Real-Time Polymerase Chain Reaction

Total RNA was isolated from CD4^+^  T cells obtained from healthy controls and patients with SLE using TRIzol (Invitrogen, USA) and 1 *μ*g total RNA reverse transcribed into cDNA using RevertAid First Strand cDNA Synthesis Kit (Fermentas, Canada). Quantitative real-time PCR was performed in duplicate using SYBR Premix Ex Tag kit (Takara, Japan) in a Rotor-Gene 3000 thermocycler. Primers used included

HMGB1  Forward: 5′-TCACAG CCATTG CAG TACATT GAG-3′
  Reverse: 5′-GGATCT CCT TTG CCC ATG TTT AGT T-3′
CD70  Forward: 5′-CACACTCTGCACCTCACT-3′
  Reverse: 5′-CACCCACTGCACTCCAAAGA-3′
CD11a  Forward: 5′-TGAGAGCAGGCTATTTGGGTTAC-3′
  Reverse: 5′-CGGCCCATGTGCTGGTAT-3′
*β*-actin  Forward: 5′-CGCGAGAAGATGACCCAGAT-3′
  Reverse: 5′-GCACTGTGTTGGCGTACAGG-3′

### 2.6. Statistical Analysis

Results were expressed as mean + SD. Intergroup analysis was performed using Student's *t*-test. Correlation of HMGB1 mRNA with CD11a, CD70 mRNA and SLEDAI was assessed by Pearson's correlation coefficient. *P* values < 0.05 were considered significant. All analyses were performed with SPSS version 15.0 software.

## 3. Results

### 3.1. Identification of Proteins That Bound to Gadd45a

**Figure 1:**
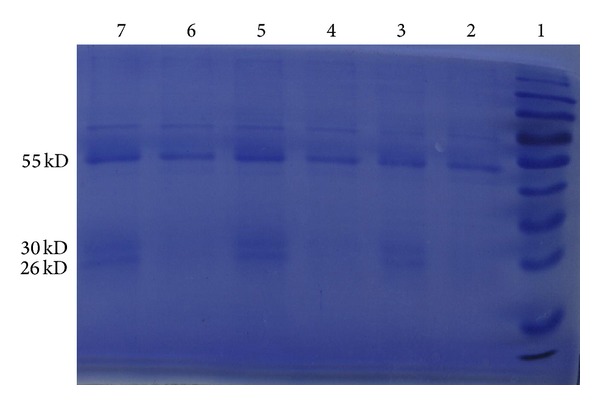
Representative gel showing protein bands immunoprecipitated with anti-human Gadd45a antibody. Lane 1: prestained markers, lanes 3, 5, and 7: immunoprecipitated with anti-Gadd45a antibody, and lanes 2, 4, and 6: immunoprecipitated with isotype-matched control IgG.

To identify proteins that bind to Gadd45a, total proteins extracted from CD4^+^  T cell were immunoprecipitated with anti-human Gadd45a antibodies, separated by SDS-PAGE, and stained with Coomassie blue. Anti-Gadd45a antibodies but not isotype-matched control immunoprecipitated two bands with MW of ~30 and ~26 kDa (Figure 1). A protein band with MW ~55 kDa was detected by both anti-Gadd45a antibodies and control IgG. These bands were excised, subjected to in-gel trypsin digestion, and subjected to Tandem MS. Data obtained were analyzed against the Gene Ontology annotations to proteins in the UniProt Knowledgebase using Mascot 2.3.02 software. (Proteins listed in Table 2 are MW of ~30 KDa and ~26 KDa proteins precipitated by anti-Gadd45a antibodies and MW ~55 KDa proteins which are different proteins between the anti-Gadd45a antibodies precipitation group and control antibody precipitation group.)

### 3.2. Gene and Protein Expression of HMGB1 in CD4^+^  T Cells from SLE Patients with Active Disease

**Figure 2:**
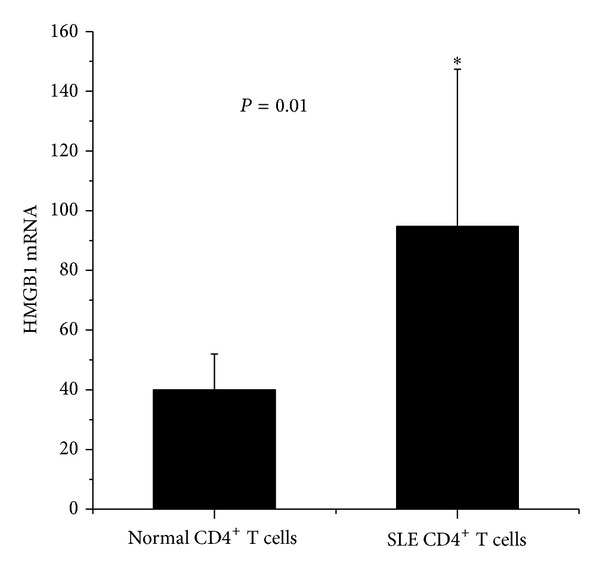
Real-time PCR quantification of HMGB1 mRNA in CD4^+^  T cells. HMGB1 mRNA in CD4^+^  T cells isolated from patients with SLE and healthy controls was determined by quantitative real-time PCR. Results are normalized to *β*-actin and presented as mean + SD.

**Figure 3:**
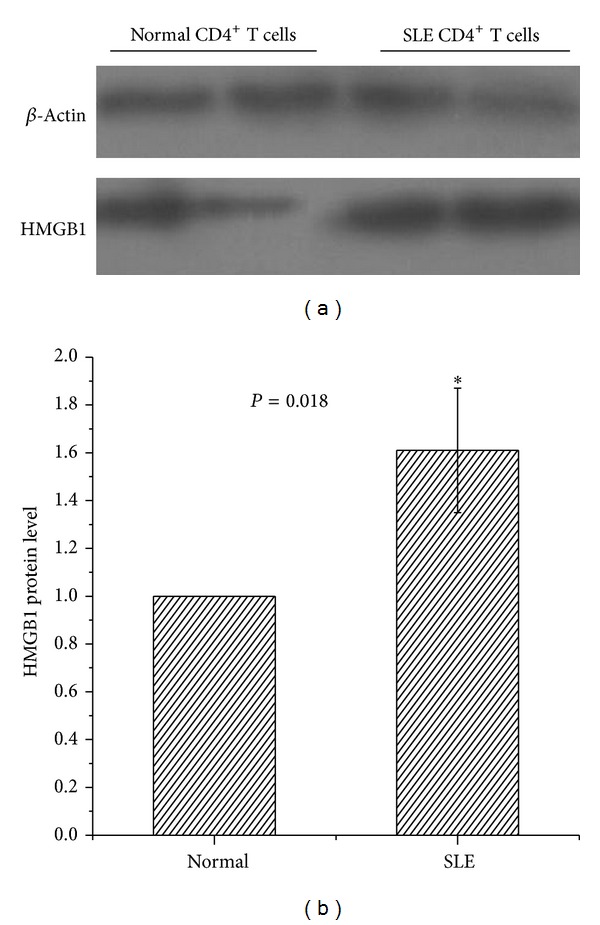
Protein expression of HMGB1 in CD4^+^  T cells using western blot. (a) Representative western blot showing protein expression of HMGB1 in CD4^+^  T cells isolated from patients with SLE and healthy controls. (b) Bar chart showing the mean intensity of the bands as determined by densitometric scan, normalized to the house-keeping gene, *β*-actin.

HMGB1 was identified as the protein that bound to Gadd45a by immunoprecipitation. Elevated expression was confirmed in PBMC and serum in SLE patients in previous studies [10–12]. In addition, it may interact with various proteins, including methy-CpG binding protein2 (MeCP2) [13]. So we compared HMGB1 mRNA levels in CD4^+^  T cells from SLE patients and healthy controls. Gene and protein expression of HMGB1 was significantly increased in SLE CD4^+^  T cells compared to CD4^+^  T cells isolated from healthy subjects (*P* = 0.01 and *P* = 0.018, resp.) (Figures 2, 3(a), and 3(b)). 

**Figure 4:**
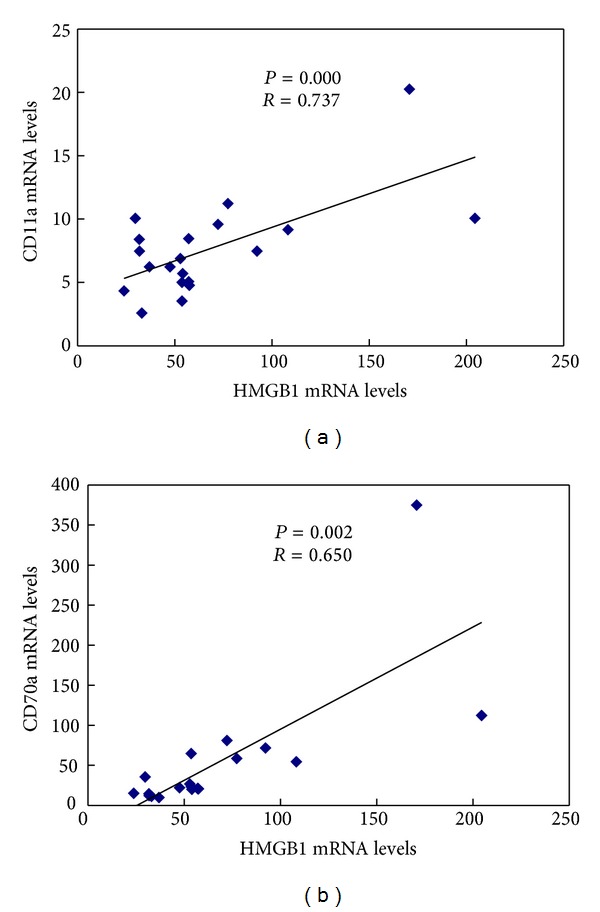
Correlation between HMGB1 mRNA and CD11a and CD70 mRNA. Gene expression of HMGB1, CD11a, and CD70 was assessed by real-time PCR. Correlation between HMGB1 mRNA and (a) CD11a mRNA and (b) CD70 mRNA is shown for CD4^+^  T cells in SLE patients and controls.

**Figure 5:**
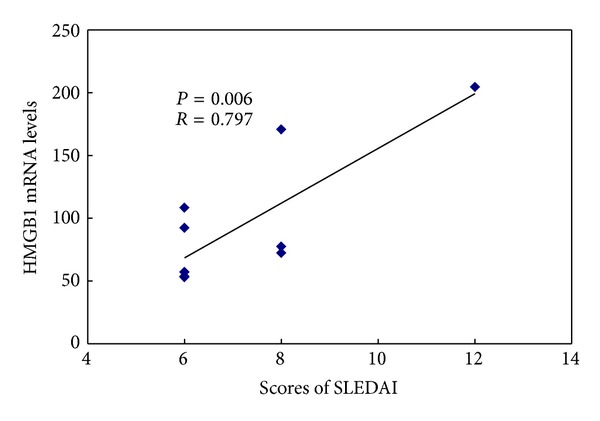
Correlation between HMGB1 mRNA and SLEDAI. Lupus disease activity was quantified by the SLE Disease Activity Index (SLEDAI). Correlation between HMGB1 mRNA in lupus CD4^+^  T cells and SLEDAI for lupus patients is shown.

Gene expression of HMGB1 was significantly correlated with CD11a and CD70 mRNA in CD4^+^  T cell (*R* = 0.737, *P* = 0.000; *R* = 0.650, *P* = 0.002, resp.) (Figures 4(a) and 4(b)). Furthermore, we analyzed the correlation between HMGB1 mRNA and lupus disease activity as measured by the SLEDAI. A positive correlation was also found between HMGB1mRNA and SLEDAI (*R* = 0.797, *P* = 0.006) (Figure 5).

## 4. Discussion

Over the past two decades, global DNA hypomethylation and gene-specific DNA demethylation have been observed in T cells in patients with idiopathic lupus and drug-induced lupus. Gadd45a (Gadd45a/Gadd45) is a nuclear protein that plays an important role in the maintenance of genomic stability, DNA repair, and suppression of cell growth. In our previous studies, we have demonstrated that Gadd45a contributes to autoimmune diseases by promoting DNA demethylation [5]. In this study, we identified proteins that bind to Gadd45a in SLE CD4^+^  T cells that may assist in DNA demethylation. Using LS-MS/MS, we identified HMGB1 as one of the proteins that bind to Gadd45a. Gene and protein expression of HMGB1 was increased in SLE CD4^+^  T cells compared to control CD4^+^  T cells. HMGB1 is a nuclear DNA binding protein secreted by monocytes and macrophages and plays a critical role in innate immunity, inflammation, and sterile injury [14]. Serum HMGB1 levels are increased in SLE patients and its expression is increased in skin lesions in lupus patients [10–12, 15]. 

HMGB1 interacts with numerous proteins such as methyl-CpG binding protein-2 (MeCP2), a protein that participates in the recognition of methylated DNA in CpG islands [13]. In this study, we demonstrated that gene and protein expression of HMGB1 in CD4^+^  T cells is significantly increased in SLE patients compared to healthy controls thereby corroborating previous studies. Furthermore, HMGB1 mRNA in CD4^+^  T cells is correlated with SLEDAI for SLE patients. Given that CD4^+^  T cells promote inflammation and autoimmunity, it is plausible to suggest that HMGB1 contributes to the pathogenesis of SLE [16]. 

CD11a plays a key role in cellular adhesion and costimulatory signaling. Its expression is increased in T cells consequent to hypomethylation and correlates with the development of T-cell autoreactivity in SLE patients [3, 4]. CD70 is a methylation-sensitive gene present in T cells. Costimulatory signals mediated through CD70-CD27 interactions regulate B-cell activation and autoantibody production, thereby highlighting the role of CD70 in the pathogenesis of SLE. In this study, we demonstrated that HMGB1 mRNA is significantly correlated with CD11a and CD70 mRNA.

In conclusion, we have demonstrated that HMGB1 binds to Gadd45a and its increased gene expression which is associated with a concomitant increase in CD11a and CD70 mRNA may be involved in DNA demethylation in CD4^+^  T cells, thus contributing to the pathogenesis of SLE. Further studies are currently ongoing to delineate the function of HMGB1 and its underlying mechanism contributing to DNA demethylation in CD4^+^  T cells during pathogenesis of SLE. 

## Figures and Tables

**Table 1:** Patient demographics and treatment.

Patient	Age/gender	SLEDAI	Treatment
1	49/F	12	None
2	38/F	6	Pred 20 mg/d
3	23/F	6	None
4	35/F	6	Pred 5 mg/d
5	15/F	8	None
6	34/F	6	None
7	24/F	8	None
8	25/F	6	None
9	22/F	8	Pred 30 mg + CTX
10	20/F	6	Pred 10 mg

F: female; SLEDAI: Systemic Lupus Erythematosus Disease Activity Index; Pred: Prednisone; CTX: Cyclophosphamide.

**Table 2:** Proteins identified by LS-MS/MS.

Protein mass	Description
	~26 KDa, ~30 KDa
**25049.23**	**HMGB1 High mobility group protein B1**
29807.14	RPS4X 40S (ribosomal protein S4, X isoform)
27898.79	YWHAZ 14-3-3 protein zeta/delta
14126.95	HIST1H2AB; HIST1H2AE; HIST1H2AD; HIST1H2AG; HIST1H2AK; HIST1H2AI; HIST1H2AL; HIST1H2AM; HIST1H2AJ Histone H2A type 1-B/E
38837.09	HNRNPA1 Isoform A1-B of Heterogeneous nuclear ribonucleoprotein A1
28876.08	PSME1 Proteasome activator complex subunit 1
32179.71	HNRNPCL1 Heterogeneous nuclear ribonucleoprotein C-like 1
36201.46	GAPDH Glyceraldehyde-3-phosphate dehydrogenase
18885.87	RPS10 40S ribosomal protein S10
33059.24	SLC25A5 ADP/ATP translocase 2
37463.75	HNRNPA2B1 Isoform B1 of Heterogeneous nuclear ribonucleoproteins A2/B1
15215.62	PFN1 Profilin-1
25119.3	IGLC2; IGLV2-14 IGL@ protein
24820.32	RAB27B Ras-related protein Rab-27B
22449.98	DRAP1 Isoform 1 of Dr1-associated corepressor
32867.15	TNFAIP8L3 Tumor necrosis factor alpha-induced protein 8-like protein 3
16982.46	LYZ Lysozyme C
31881.8	STOM Erythrocyte band 7 integral membrane protein
24764.75	SNRPB Isoform SM-B′ of Small nuclear ribonucleoprotein-associated proteins B and B′
19545.99	SRSF3 Serine/arginine-rich splicing factor 3
17707.86	RPS18 40S ribosomal protein S18
38805.89	ANXA2P2 Putative annexin A2-like protein
26688.86	AK2 Isoform 1 of Adenylate kinase 2, mitochondrial
21950.62	IMP3 U3 small nucleolar ribonucleoprotein protein IMP3
22113.26	RPS7 40S ribosomal protein S7
30729.42	ASB13 Isoform 1 of Ankyrin repeat and SOCS box protein 13

	~55 KDa
52076.78	PPP2R2B Isoform 1 of Serine/threonine-protein phosphatase 2A 55
52324.86	CAP1 Isoform 1 of Adenylyl cyclase-associated protein 1
56456.49	EEF1G cDNA FLJ56389, highly similar to Elongation factor 1-gamma
52586.54	EIF3E Eukaryotic translation initiation factor 3 subunit E
